# Genome-Wide Analysis of Exocyst Complex Subunit Exo70 Gene Family in Cucumber

**DOI:** 10.3390/ijms241310929

**Published:** 2023-06-30

**Authors:** Liu Liu, Chaoheng Gu, Jiahao Zhang, Jingyu Guo, Xiaolan Zhang, Zhaoyang Zhou

**Affiliations:** Beijing Key Laboratory of Growth and Developmental Regulation for Protected Vegetable Crops, Department of Vegetable Sciences, China Agricultural University, Beijing 100193, China; b20193170868@cau.edu.cn (L.L.); s20213172762@cau.edu.cn (C.G.); zjh7292@cau.edu.cn (J.Z.); guojingyu_96@163.com (J.G.); zhxiaolan@cau.edu.cn (X.Z.)

**Keywords:** cucumber, *Exo70*, systematical analyses, expression analyses

## Abstract

Cucumber (*Cucumis sativus* L.) is an important vegetable worldwide, but its yield is affected by a wide range of pathogens and pests. As the major subunit of the exocyst complex, the roles of Exo70 members have been shown in *Arabidopsis* and rice, but their function are unknown in cucumber. Here, we identified 18 CsExo70 members in cucumber, which were divided into three groups (Exo70.1–Exo70.3) and nine subgroups (Exo70A–Exo70I) based on the phylogenetic tree. Subsequently, systematical analyses were performed, including collinearity, gene structure, *cis*-acting elements, conserved motifs, expression patterns, and subcellular localization. Our results showed that *CsExo70* genes were generally expressed in all tissues, and *CsExo70C1* and *CsExo70C2* were highly expressed in the stamen. Moreover, the expression levels of most *CsExo70* genes were induced by *Pseudomonas syringae* pv. *lachrymans* (*Psl*) and *Fusarium oxysporum* f. sp. *cucumerinum* Owen (*Foc*), especially *CsExo70E2* and *CsExo70H3*. In addition, these CsExo70s displayed similar location patterns with discrete and punctate signals in the cytoplasm. Together, our results indicate that CsExo70 members may be involved in plant development and resistance, and provide a reference for future in-depth studies of *Exo70* genes in cucumber.

## 1. Introduction

Secretion is a vital cellular process and is responsible for the transport of newly synthesized materials, ending with the exocytosis event at the plasma membrane (PM) in eukaryotes [1]. De novo cargoes of a vast array of proteins, including PM proteins, signaling peptides, or small RNAs, are transported to the PM or extracellular space by vesicles [2,3,4]. Prior to soluble N-ethylmaleimide-sensitive factor (NSF) attachment protein receptor (SNARE)-mediated membrane fusion, the first attachment of secretory vesicles to the target PM is mediated by the exocyst complex [1,5], which was firstly identified in budding yeast (*Saccharomyces cerevisiae*). The exocyst complex consists of eight subunits: SEC3, SEC5, SEC6, SEC8, SEC10, SEC15, Exo70, and Exo84 [6]. Of note, it has been suggested that SEC3 and Exo70 are activated by Rho GTPases and create the initial contact points between vesicle and PM as the major tethers [7,8,9].

Although only one copy of the Exo70 exists in *Saccharomyces*, *Drosophila*, and *Caenorhabditis*, there are multiple copies of Exo70 in plants [10], such as 13 paralogs in moss (*Physcomitrella patens*), 23 paralogs in *Arabidopsis thaliana*, 41 paralogs in rice (*Oryza sativa*), and 23 paralogs in poplar (*Populus trichocarpa*). These Exo70 members are divided into three clades (Exo70.1 to Exo70.3) and are broadly involved in multiple biological processes in plants [11,12,13]. For instance, *Arabidopsis* Exo70A1 is required for polar auxin transport in a PIN1/2-dependent pathway. The mutation of *Exo70A1* leads to altered auxin distribution, loss of apical dominance, and shorter root hairs [14,15]. Additionally, *Exo70A1/2* and *Exo70C1/2* function as regulators in sexual reproduction; the *exo70a1* mutant exhibits fertility defects, and *Exo70A2* mutation results in impaired pollen germination and pollen tube growth [14,16], while Exo70C1/2 are involved in the regulation of optimal tip growth of pollen tubes [17]. Furthermore, the *Arabidopsis* Exo70H4 is involved in trichome cell wall maturation, and its paralog is also highly expressed in cucumber fruit trichomes, suggesting a conserved function of Exo70H in trichome development [18,19]. The Exo70 members also participate in plant immunity. For instance, the expression of *Exo70H1* and *Exo70B2* was up-regulated after pathogen incubation in *Arabidopsis* [20]. *Exo70B2* and its homolog *Exo70B1* are both required for pattern-triggered immunity (PTI). Accordingly, *exo70B1-3* and *exo70B2-1* mutants both display enhanced susceptibility to *Pseudomonas syringae* pv *tomato* (*Pst*) DC3000 [21,22,23]. Interestingly, the loss function of *Exo70B1* also leads to an activation of TIR-NBS2 (TN2), thus enhancing resistance to multiple pathogens in TN2-dependent autoimmunity [24]. Recent studies showed that Exo70B1/2 regulated FLAGELLIN SENSING 2 (FLS2) accumulation at the PM, which is required for the outcome of immune responses in *Arabidopsis* [25]. Moreover, OsExo70B1 interacted with Chitin Elicitor Receptor Kinase 1 (CERK1) and was involved in resistance to rice blast fungus *Magnaporthe oryzae* (*M. oryzae*) [26], but the mechanism is still largely unknown.

Cucumber (*Cucumis sativus* L.) is an important vegetable crop of high economic and biological value [27,28]. Along its evolution, the cucumber has diverse sex patterns and has become a model plant for studying plant sex determination [29,30]. Moreover, cucumber usually bears fruits of various sizes, shapes, and colors [31,32,33,34], covered with tubercules, trichomes, and a thick cuticle [35,36]. However, its production is substantially affected by the frequent incidence of multiple diseases. For instance, cucumber bacterial angular leaf spot disease (ALS) is caused by *Pseudomonas syringae* pv. *lachrymans* (*Psl*), which mainly infects the leaves and results in limited vein and necrotic tissues [37,38]. In addition, cucumber fusarium wilt, one of the major fungal diseases, is a soil-borne disease caused by *Fusarium oxysporum* f. sp. *cucumerinum* Owen (*Foc*), which usually leads to a decrease in photosynthesis capacity and loss of yield [39,40]. The Exo70s have been studied in multiple plant species [41,42,43,44], but not cucumber.

At present, genome-wide identification and gene expression analysis are effective approaches to study the classification and potential functions of gene family members, which provides a foundation for further gene function identification [43]. In this study, we aim to identify the CsExo70 members in cucumber. A genome-wide analysis of CsExo70 members was performed, including the system evolution, collinearity, gene structure, *cis*-acting elements, conserved motifs, subcellular localization, expression patterns in different tissues, and treatment with pathogens. This study will provide a reference for further investigating the functions of the *CsExo70* gene family in the future.

## 2. Results

### 2.1. Identification and Characterization of CsExo70 Family in Cucumber

**Figure 1:**
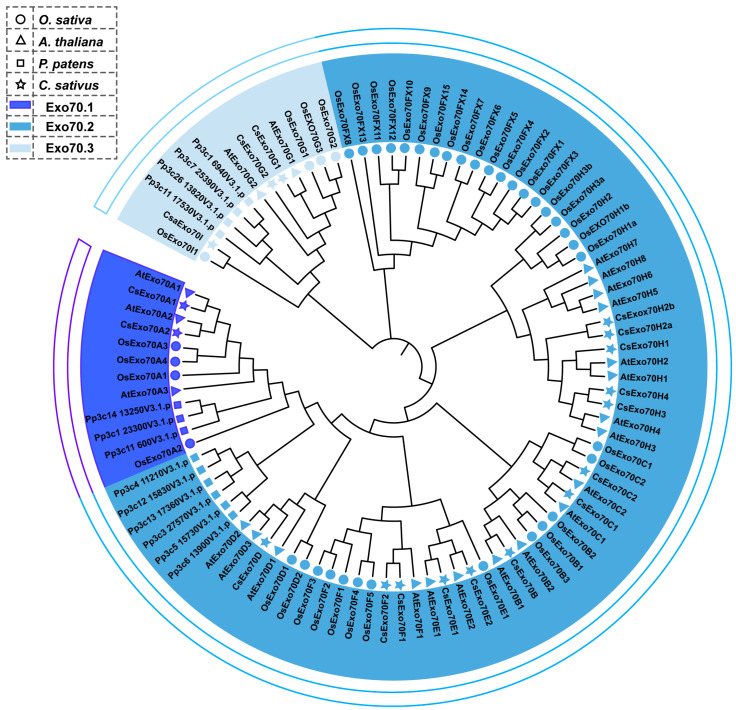
Phylogenetic analysis of Exo70 family. The circle, triangle, square, and star represent Exo70s in *Oryza sativa* (*O. sativa*)*, Arabidopsis thaliana* (*A. thaliana*), *Physcomitrella patens* (*P. patens*), and *Cucumis sativus* (*C. sativus*), respectively.

In this study, 18 CsExo70 members were identified, which have conserved Exo70 domains (Figure S1). To explore the evolutionary relationships of Exo70s among different species, the full-length sequences of Exo70s of cucumber, *Arabidopsis*, rice, and moss were used to generate a phylogenetic tree. As shown in Figure 1, all CsExo70 members were divided into three groups (Exo70.1 to Exo70.3) and nine subgroups (Exo70A to Exo70I), and named according to the phylogenetic tree. Except for CsExo70B, CsExo70D, and CsExo70I, other members have several homologs in cucumber.

Additionally, the physical and chemical characteristics of CsExo70s were predicted in Table 1, including the length of the coding sequence (CDS) and amino acid (AA) sequence, molecular weight (MW), and isoelectric point (PI) values. In detail, the protein length ranged from 582 (CsExo70H4) to 704 (CsExo70C2) amino acids, MW varied from 66,156.59 Da (CsExo70H4) to 81346.62 Da (CsExo70C2), and the PI value varied from 4.94 (CsExo70E1) to 8.87 (CsExo70G1).

### 2.2. Chromosomal Localization and Collinearity Analysis of CsExo70 Genes in Cucumber

**Figure 2:**
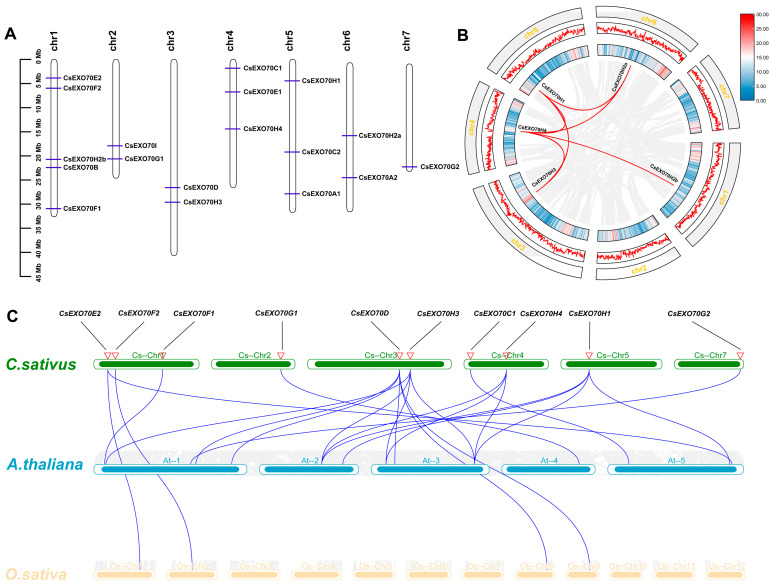
Chromosomal location and collinearity analysis of *Exo70*s in cucumber. (**A**) Locations of *CsExo70* genes in seven chromosomes of cucumber. (**B**) Genome-wide synteny analysis of *Exo70s* in *C. sativus* genome. Red lines indicate the paralogous genes. (**C**) Genome-wide synteny analysis of *Exo70s* between *C. sativus* and *A. thaliana*, and *C. sativus* and *O. sativa* genomes. Blue lines represent the orthologous genes, the red triangles represent different gene pairs.

According to the physical location of all *CsExo70* genes in the *C. sativus* genome, the gene chromosomal distribution was drawn. As shown in Figure 2A, 18 *CsExo70* genes were distributed over the seven cucumber chromosomes. There were five genes located on chromosome 1, three genes on chromosomes 4 and 5, two genes on chromosomes 2, 3, and 6, and only one gene on chromosome 7. Moreover, some *CsExo70* genes had adjacent locations, such as *CsExo70E2* and *CsExo70F2*, *CsExo70B* and *CsExo70H2b*, and *CsExo70G1* and *CsExo70I*, but no tandem-duplicated *CsExo70* genes were found.

We further explored the segmental duplication events of *CsExo70* genes via collinearity analysis in the *C. sativus* genome. As shown in Figure 2B, six pairs of segmentally duplicated genes were identified in the *C. sativus* genome: *CsExo70H1/CsExo70H4*, *CsExo70H1/CsExo70H2a*, *CsExo70H1/CsExo70H3*, *CsExo70H2a/CsExo70H4*, *CsExo70H3/CsExo70H4*, and *CsExo70H4/CsExo70H2b*, indicating that *CsExo70H* members were amplified via segmental duplication events. Furthermore, to better understand the gene amplification pattern during evolution, collinearity analysis among cucumber, *Arabidopsis,* and rice was performed (Figure 2C). In total, 23 gene pairs were identified. There were 19 pairs between *C. sativus* and *A. thaliana*, and 4 pairs between *C. sativus* and *O. sativa*, suggesting the *Exo70* genes of *C. sativus* had higher synteny with *A. thaliana* than *O. sativa* during the evolution.

### 2.3. Gene Structure and Conserved Motif Analysis of CsExo70 Genes

**Figure 3:**
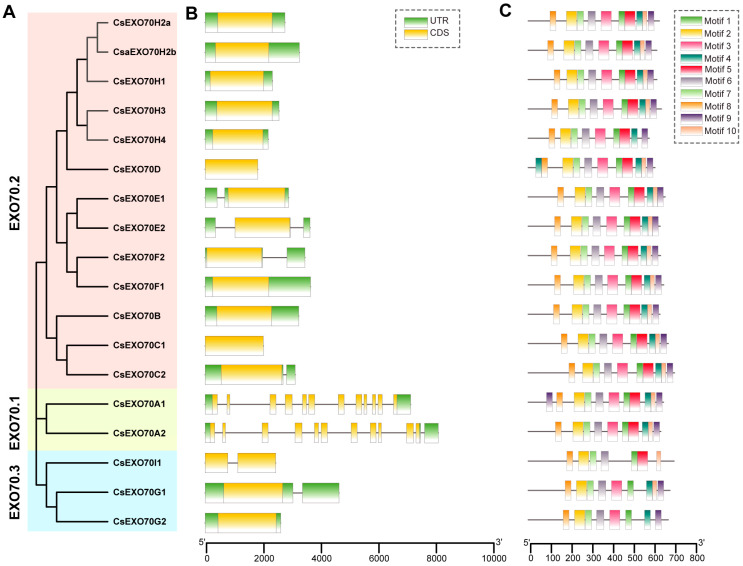
Gene structure and conserved motifs of Exo70 family in cucumber. (**A**) The phylogenetic tree of Exo70 members in cucumber. (**B**) Gene structures of *CsExo70*s. The yellow and green boxes represented coding sequence (CDS) and untranslated region (UTR), respectively. The black lines indicated introns. (**C**) Conserved motifs of CsExo70 proteins; the ten motifs are displayed in different colors.

The gene structure and sequence characteristics of *Exo70* genes were further investigated in cucumber. Gene structure analysis showed that most genes had 5′Untranslated Region (UTR) or 3′UTR. Among them, genes in Exo70.2 and Exo70.3 clades displayed a similar structure with 1–2 exons and 0–2 introns, while the genes in the Exo70.1 clade consisted of 11–12 exons and 11 introns (Figure 3B). These results suggest that genes in the same clade have a similar structure and that functional differentiation may exist between different clades.

Moreover, the conserved motifs in CsExo70s were analyzed via MEME. A total of 10 conserved motifs were identified in all CsExo70 members. Except for Exo70H4, Exo70D, and Exo70A1, the members in Exo70.1 and Exo70.2 clades had all of these motifs, while Exo70I, Exo70G1, and Exo70G2 in the Exo70.3 clade lacked three, one, and two motifs, respectively. Although the functions of motifs 1–10 have not been revealed, these motifs were mainly located on the C-terminal, which contains the conserved Exo70 domain that determines the function of these proteins. Thus, it speculated that these members of the Exo70.3 clade may have different functions from that in other clades.

### 2.4. Analysis of Cis-Acting Elements on CsExo70 Promotors

**Figure 4:**
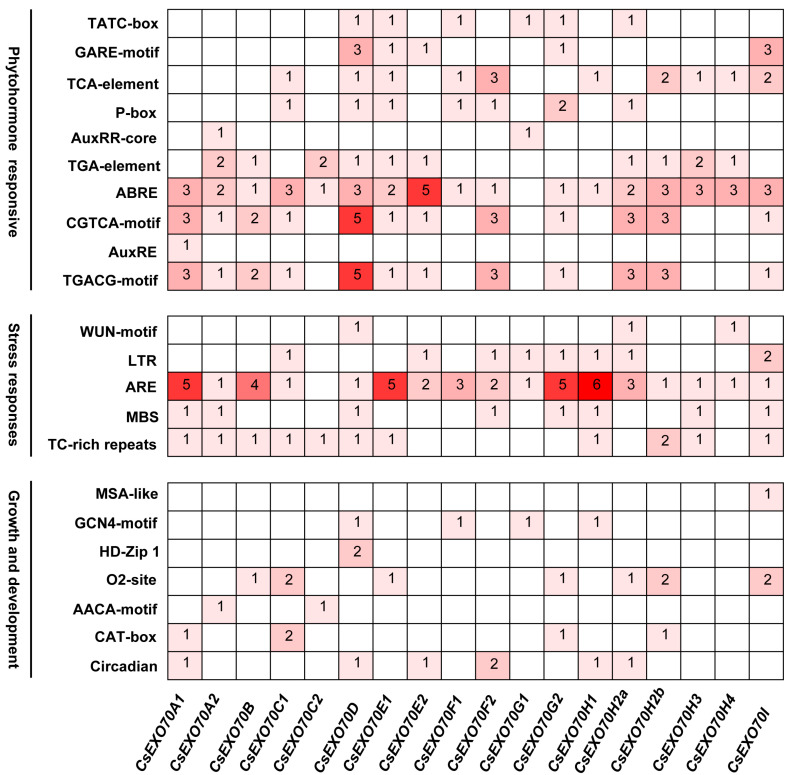
*Cis*-acting elements in the promotors of *Exo70* genes in cucumber. The colors indicate the different *cis*-elements numbers. Values indicate the statistical number of *cis*-elements.

To study the *CsExo70* gene expression regulation, the promoter sequences (2000 bp upstream of the start codon) of 18 *CsExo70*s were analyzed. The major *cis*-acting elements were identified (Figure 4). The most *cis*-acting elements were relevant to phytohormones, including related to responses of methyl jasmonate (TGACG motif/CGTCA motif), abscisic acid (ABRE motif), gibberellin (P-box/GARE motif/TATC box), auxin (AuxRE/AuxRR/ TGA motif), and salicylic acid (TCA motif). Among these, the abscisic acid-response element (ABRE) could be found on all *CsExo70* promoters, except *CsExo70G1*. Moreover, these *CsExo70* promoters contain stress-responsive elements, including anaerobic-induction elements (ARE), defense-responsive elements (TC-rich repeats), low-temperature responsive (LTR) elements, drought-induction elements (MBS), and wound-responsive elements (WUN motif). *Cis*-acting elements relevant to growth and development were also found, including circadian control, meristem expression (CAT box), endosperm-specific expression (AACA motif/GCN4 motif), zein metabolism regulation (O_2_ site), mesophyll cell differentiation (HD-Zip), and cell cycle regulation (MSA-like). These results suggest that *CsExo70* genes may be involved in multiple biological processes.

### 2.5. Expression Patterns of CsExo70 Genes in Cucumber

**Figure 5:**
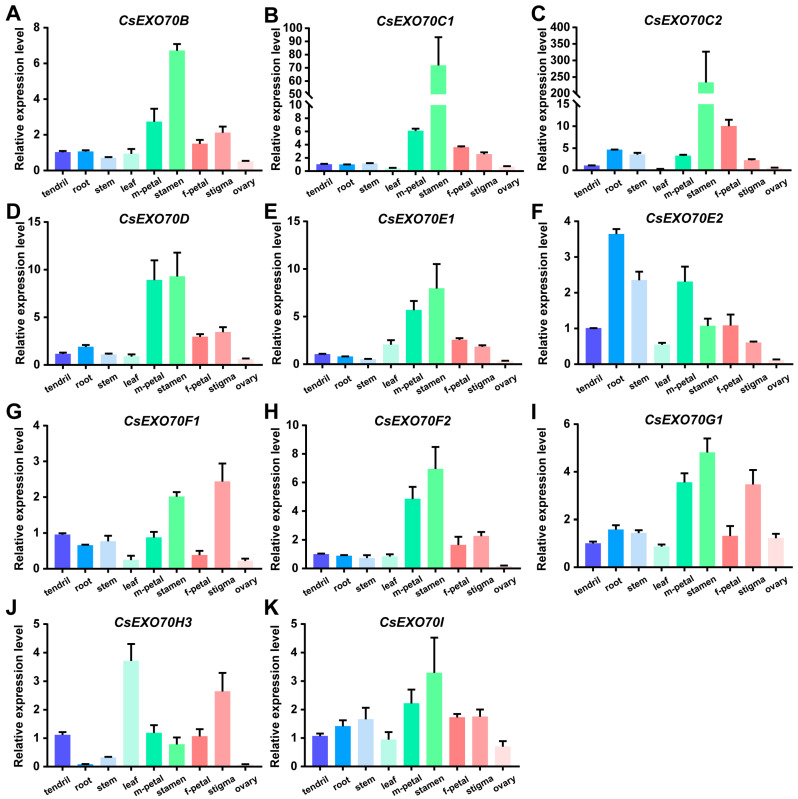
Expression patterns of *CsExo70B* (**A**), *CsExo70C1* (B), *CsExo70C2* (**C**), *CsExo70D* (**D**), *CsExo70E1* (**E**), *CsExo70E2* (**F**), *CsExo70F1* (**G**), *CsExo70F2* (**H**), *CsExo70G1* (**I**), *CsExo70H3* (**J**), and *CsExo70I* (**K**) in cucumber tissues. The cucumber *UBQ* gene was used as an internal standard. m, male; f, female.

To investigate tissue-specific expression, the expression patterns of all 18 *CsExo70* genes were examined by quantitative RT-PCR (qRT-PCR) in different cucumber tissues during the reproductive growth stage, including tendril, root, stem, leaf, male-flower petal, stamen, female-flower petal, stigma, and ovary (Figure 5). Among 18 *CsExo70*s, seven genes (*CsExo70A1*, *CsExo70A2*, *CsExo70G2*, *CsExo70H1*, *CsExo70H2a*, *CsExo70H2b*, and *CsExo70H4*) have lower transcript levels and were almost not detected in all tissues. Alternatively, these genes were expressed in other development stages. Interestingly, most of the remaining 11 genes displayed a similar tissue expression pattern with the highest transcripts in the stamen, especially for *CsExo70C1* and *CsExo70C2* (Figure 5B,C), consistent with *Exo70C* homologous in *Arabidopsis*, rice, and cotton [13,17,41], indicating that *Exo70C* members may have a conserved function in the regulation of stamen fertility. Moreover, *CsExo70F1*, *CsExo70G1*, and *CsExo70H3* were highly expressed in stigma (Figure 5G,I,J), the transcript of *CsExo70E2* was higher in root and stem, and *CsExo70H3* was highly expressed in the leaf (Figure 5F,J). These results suggest that *CsExo70* members may regulate the growth and development of multiple tissues.

### 2.6. CsExo70 Gene Expression in Response to Different Pathogens

**Figure 6:**
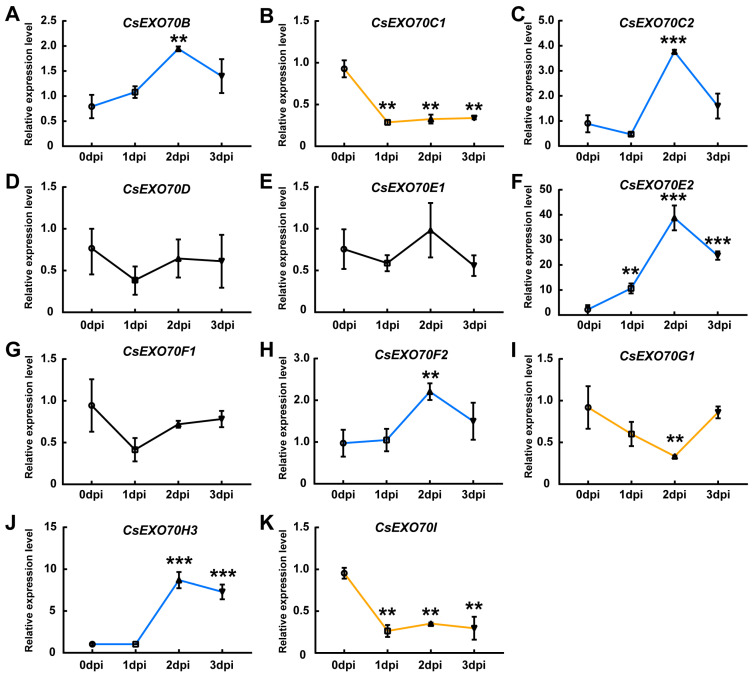
*Pseudomonas syringae* pv. *lachrymans* (*Psl*)-triggered *CsExo70* expression in cucumber. (**A**–**K**) The transcript levels of *CsExo70B* (**A**), *CsExo70C1* (**B**), *CsExo70C2* (**C**), *CsExo70D* (**D**), *CsExo70E1* (**E**), *CsExo70E2* (**F**), *CsExo70F1* (**G**), *CsExo70F2* (**H**), *CsExo70G1* (**I**), *CsExo70H3* (**J**), and *CsExo70I* (**K**) were detected after *Psl* infection. Leaves of four-week-old cucumber seedlings were treated with *Psl*, and harvested at 0, 1, 2, 3 days after inoculation (dpi) for expression analysis. *CsExo70* transcripts were quantified by qRT-PCR using *UBQ* as the internal standard. Significant differences are indicated by asterisks (** *p* < 0.01, *** *p* < 0.001, Student’s *t* test). Blue and yellow lines represent increased or decreased genes, respectively; the black line indicates genes without a significant change.

Previous studies have demonstrated that the transcripts of *Exo70B2* and *Exo70H1* were induced by bacterial elongation factor-TU epitope elf18 in *Arabidopsis* [20]. In rice, the transcript of *OsExo70B1* was increased after the pathogen-associated molecular pattern (PAMP) and *M. oryzae* treatment [26]. To investigate pathogen-triggered *CsExo70* expression in cucumber, the expression levels of these genes at different time points after *Psl* treatment were detected. We observed that the expression of *CsExo70E2* was about 40-fold higher at 2 days post inoculation (dpi) than that at 0 dpi (Figure 6F). The expression of *CsExo70H3* was also significantly increased to 7–8-fold at 2–3 dpi (Figure 6J), while the expression of *CsExo70B*, *CsExo70C2*, and *CsExo70F2* was slightly up-regulated at 2 dpi (Figure 6A,C,H). In contrast, *CsExo70C1, CsExo70G1,* and *CsExo70I* were down-regulated after *Psl* infection (Figure 6B,I,K), and the expression levels of *CsExo70D, CsExo70E1,* and *CsExo70F1* were unaffected after *Psl* treatment (Figure 6D,E,G). These results indicate that some *CsExo70* genes may play essential roles in resistance to *Psl* in cucumber.

**Figure 7:**
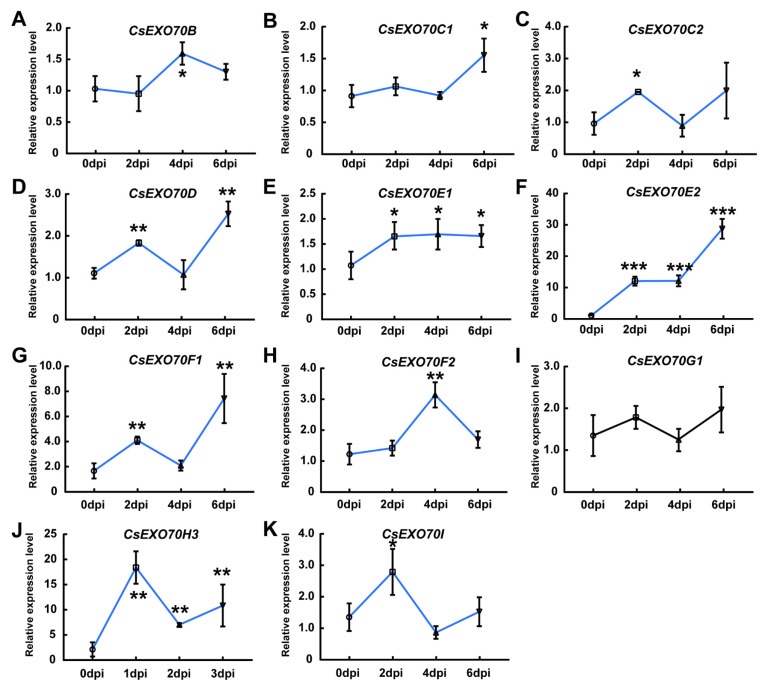
Expression analysis of *CsExo70B* (**A**), *CsExo70C1* (**B**), *CsExo70C2* (**C**), *CsExo70D* (**D**), *CsExo70E1* (**E**), *CsExo70E2* (**F**), *CsExo70F1* (**G**), *CsExo70F2* (**H**), *CsExo70G1* (**I**), *CsExo70H3* (**J**), and *CsExo70I* (**K**) after *Fusarium oxysporum* f. sp. *cucumerinum* Owen (*Foc*) treatment. One-week-old cucumber seedlings were treated with *Foc* and roots were collected at 0, 2, 4, and 6 days after inoculation (dpi). Values are means ± sd of three biological replicates. Significant differences between 0 dpi and other time points are indicated by asterisks (* *p* < 0.05, ** *p* < 0.01, *** *p* < 0.001, Student’s *t* test). The blue lines represent up-regulated genes, and the black line indicates genes without a significant change at any time point.

To verify whether *CsExo70* genes are also responsive to fungal pathogen, *Foc*-triggered *CsExo70* expression was examined. Similar to *Psl* treatment, the expressions of *CsExo70E2* and *CsExo70H3* were also significantly increased in response to *Foc* (Figure 7F,J). The same results were also observed for *CsExo70B*, *CsExo70C2*, and *CsExo70F2* expression levels at 2 dpi or 4 dpi after *Foc* treatment (Figure 7A,C,H). Unlike that after *Psl* inoculation, the expression of *CsExo70C1* and *CsExo70I* was up-regulated at 6 and 2 dpi, respectively (Figure 7B,K), while the expression of *CsExo70G1* was unchanged in response to *Foc* (Figure 7I). Furthermore, the expression levels of *CsExo70D* and *CsExo70F1* were instantaneously increased at 2 dpi, then decreased to normal levels at 4 dpi, and increased at 6 dpi (Figure 7D,G). *CsExo70E1* was weakly up-regulated after *Foc* infection, and *CsExo70G1* expression was unchanged by *Foc* treatment (Figure 7E,I). These results indicate that the expression of most *CsExo70* genes was induced by pathogen treatment, especially *CsExo70E2* and *CsExo70H3*, which may play important roles in plant defense against both fungal and bacterial diseases in cucumber.

### 2.7. Subcellular Localization Analysis

**Figure 8:**
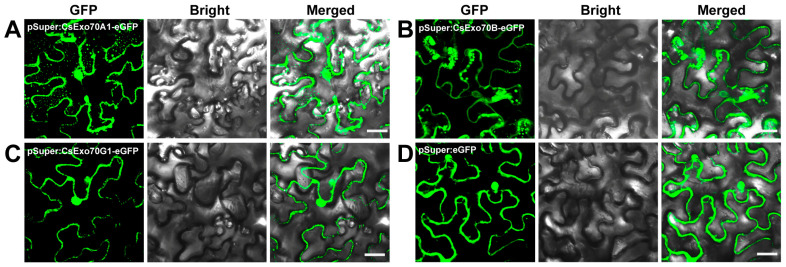
Subcellular localizations of CsExo70A1 (**A**), CsExo70B (**B**), and CsExo70G1 (**C**) in *Nicotiana benthamiana* (*N*. *benthamiana*). (**D**) The empty vector was used as a control. These indicated structures were transiently expressed in *N*. *benthamiana* leaves. Bar, 40 μm.

To examine the subcellular location of CsExo70 members, we randomly selected one member from each clade for analysis. CsExo70A1, CsExo70B, and CsExo70G1 were fused with the GFP (green fluorescent protein) and transiently expressed in *Nicotiana benthamiana* (*N. benthamiana*) leaves. CsExo70A1 and CsExo70G1 were mainly localized in the cytoplasm and nucleus, and CsExo70B was widely distributed in PM, cytoplasm, and nucleus. Furthermore, the fluorescence signals in the cytoplasm of CsExo70A1, CsExo70B, and CsExo70G1 were discrete and punctate (Figure 8), consistent with the localization of Exo70B1 and Exo70H3 in *Arabidopsis* and rice [26,45,46,47], indicating a similar localization of Exo70s in different species.

## 3. Discussion

Previous studies have shown that the Exo70 subunit has expanded to a larger family in plants compared to that in yeast and animals [13,14,41], indicating the amplification of the *Exo70* gene in plants. In this study, 18 cucumber Exo70 members were identified by sequence BLASTp search and phylogenetic analysis (Figure 1), and divided into three clades (Exo70.1 to Exo70.3) and nine subclades (Exo70A to Exo70I) based on the different gene structures (Figure 3), which was consistent with previous studies in other species [13,42,43,44], suggesting the conservation and similarity of *Exo70* gene family in the evolution among different species. Moreover, previous studies have shown that the Exo70I subclade is commonly found in monocotyledonous plants (rice and wheat) but not dicotyledonous plants (*Arabidopsis*, cotton, and grape) [13,42,43,44]. However, CsExo70I was also identified in this study, more similar to rice but not *Arabidopsis*, suggesting that the Exo70I branch also exists in dicotyledonous plants.

Tandem and segmental duplication are the main types of gene replication events [48]. Through collinear analysis, we found six segmental duplication events in the CsExo70H subclade (Figure 2B), which contained the largest numbers among the CsExo70 subclades, meaning segmental duplication was the dominant driver of duplication of *CsExo70H* genes. On the other hand, *CsExo70* genes in Exo70.1 clade contained 11–12 exons, while only one or two exons were found in Exo70.2 and Exo70.3 clade genes (Figure 3B). Moreover, it is noteworthy to observe that all ten conserved motifs were generally distributed members in Exo70.1 and Exo70.2 clades, but not in Exo70.3 clade (Figure 3C). As mentioned above, the differences in gene structures and conserved motifs may result in function differentiation.

Moreover, the *cis*-acting elements on the *CsExo70* promoters are related to phytohormones, stress, and growth and development (Figure 4). Consistent with a previous report that Exo70A1 is involved in auxin transport and PIN recycling in *Arabidopsis* [15], more auxin-related elements (AuxRE, AuxRR, TGA elements) were observed on the promoters of *CsExo70A1* and *CsExo70A2*. In addition, when plants are subjected to stresses, some transcription factors will bind the *cis*-elements to promote the expression of related genes [49,50,51]. Notably, methyl jasmonate (MeJA) is one of the vital regulatory factors in plant resistance signal transduction pathways, and the elements of MeJA-responsive (CGTCA-motif and TGACG-motif) were mainly presented in *CsExo70D* promotor; abscisic acid (ABA) is broadly involved in drought stress and salt stress [52,53]. ABRE as the major element regulated ABA-responsive gene expression [53], was distributed on almost every promoter of *CsExo70s*, with a maximum of *CsExo70E2*, suggesting the significant roles of *CsExo70D* and *CsExo70E2* in MeJA and ABA signaling pathways, respectively. Furthermore, a total of 43 AREs were found on the promotors of *CsExo70s*, implying the potential function of *CsExo70s* in oxidative responsive, especially for *CsExo70A1*, *CsExo70B*, *CsExo70E1*, *CsExo70G2*, and *CsExo70H2*. It is speculated that *CsExo70s* may play essential roles in plant development and stress responses.

A previous study has reported that Exo70C1/2 is involved in the regulation of optimal tip growth of pollen tubes [17]; the expression pattern analysis in cucumber showed that *CsExo70C1* and *CsExo70C2* were highly expressed in the stamen, which further provided support for the involvement of *Exo70C1/2* in sexual reproduction [13,17,41,54], suggesting the conservative function of Exo70 members in different plant species. On the other hand, pathogens affect cucumber growth and development and cause a variety of diseases, resulting in a decrease in cucumber yield [55]. qRT-PCR results showed that most *CsExo70* genes were induced by pathogens (Figure 6 and Figure 7). Consistent with the *exo70H1* mutant being susceptible to *Pseudomonas syringae* pv. *maculicola* (*Psm*) [20], *Psl*-induced expression of *CsExo70H3* was about 10-fold higher at 2 dpi than that at 0 dpi, indicating that *CsExo70H3* may be also involved in *Psl* resistance in cucumber. Cucumber fusarium wilt is a soil-borne vascular disease, which usually infects cucumber roots [56]. Our data showed that the expression of *CsExo70E2* was about 30-fold higher at 6 dpi than 0 dpi after *Foc* treatment. These results suggested that *CsExo70E2* may play an essential role in resistance to *Foc* in cucumber.

The feature of subcellular compartments is related to their specialized biological functions [57]. In this study, CsExo70A1, CsExo70B, and CsExo70G1 all showed fluorescent signals in the cytoplasm and nucleus, and the signals were discrete and punctate, similar to previous studies [26,45,46,47]. Moreover, previous studies showed that Exo70B interacts with PM-localization receptor kinases (RKs) to regulate plant immunity [25,26]. This can explain why CsExo70B also displayed the PM signals. Given that the transcripts of *CsExo70B* are also induced by *Psl* and *Foc* infection, it is worth exploring whether Exo70B interacts with immune-related RKs to regulate pathogen resistance in cucumber.

## 4. Material and Methods

### 4.1. Plant Materials

Cucumber (*Cucumis sativus* L.) inbred line XTMC was used in this study. The cucumber seedlings at the two-true leaf stage were transplanted to a greenhouse under standard management at the China Agricultural University, Beijing. The *N. bethamiana* plants used for CsExo70 subcellular localization analysis were grown in a chamber at 24 °C with 16 h light/8 h dark.

### 4.2. Identification and Phylogenetic Tree Construction of Exo70 Family

To identify the predicted *Exo70* genes in the *C. sativus* genome, the amino acid sequences of 23 AtExo70 members were downloaded from the TAIR database (https://www.arabidopsis.org, accessed on 3 March 2023), and used as queries to BLASTp against the cucumber version 3 genome database (http://www.cucurbitgenomics.org/organism/20, accessed on 4 March 2023). All predicted CsExo70s were further verified by the Exo70 conserved domain by SMART (http://smart.emblheidelberg.de, accessed on 4 March 2023) and CDD-research (https://www.ncbi.nlm.nih.gov/Structure/bwrpsb/bwrpsb.cgi, accessed on 4 March 2023). The characteristics of CsExo70s, including the protein molecular weight (MW) and isoelectric point (*pI*), were analyzed via ExPaSy (http://web.expasy.org/protparam/, accessed on 5 March 2023).

The Exo70 protein sequences of *O. sativa* and *P. patens* were obtained from Phytozome13 website (https://phytozome-next.jgi.doe.gov/, accessed on 6 March 2023). Multiple sequence alignment analysis of Exo70s in cucumber, *Arabidopsis*, rice, and moss were performed in MEGA V7.0 software according to the Clustalw algorithm. Subsequently, the alignment was used to generate a phylogenetic tree using the neighbor-joining (NJ) method (bootstrap = 1000 repetitions).

### 4.3. Chromosomal Location and Synteny Analysis

The chromosomal locations of *CsExo70* genes were obtained through the Cucurbit Genomics Database. The *CsExo70* genes were mapped on different chromosomes and ultimately plotted using the TBtools [58] according to their physical positions. The syntenic maps of *Exo70* genes were generated and analyzed by TBtools [58] and displayed by Advanced Circos and Multiple Synteny Plot.

### 4.4. Gene Structures, Conserved Motifs, and Cis-Element Analysis

Gene structure analysis was performed by TBtools [58], and the conserved motifs of CsExo70 proteins were analyzed using Multiple Expectation Maximization for Motif Elicitation (MEME) (https://meme-suite.org/meme/tools/meme, accessed on 12 March 2023). The promoter sequences (2000 bp upstream of the start codon) were extracted using NCBI (https://www.ncbi.nlm.nih.gov/, accessed on 18 March 2023), and the transcriptional response *cis*-elements in promoters were predicted using the PlantCARE database (https://bioinformatics.psb.ugent.be/webtools/plantcare/html/, accessed on 20 March 2023) and drawn by TBtools [58].

### 4.5. RNA Extraction and Gene Expression Analysis

To determine the expression patterns of these *CsExo70* genes in different tissues, samples of roots, stems, leaves, male flowers, female flowers, stigmas, stamens, ovaries, and tendrils were collected during the reproductive growth stage for RNA extraction.

The total RNA was extracted using an Eastep^®^ Super Total RNA Extraction Kit (Promega, Madison, WI, USA) according to the manufacturer’s instructions, and then reverse-transcribed to complementary DNA (cDNA) using a FastKing gDNA Dispelling RT SuperMix Kit (Tiangen, Beijing, China). Subsequently, qRT-PCR was performed with *Taq* Pro Universal SYBR qPCR Master Mix (Vazyme, Nanjing, China) in a CFX384 Real-Time PCR System (BIO-RAD, Hercules, CA, USA). Three biological replicates and three technical replicates were performed for every *CsExo70* gene and *UBQ* gene (*CsaV3_5G031430*, an internal standard). The data were analyzed using the 2^−∆∆Ct^ method [59]. All primers used for qRT-PCR are listed in Table S1.

### 4.6. Plant Pathogen Treatment

For analysis of *Psl*-inducible gene expression, a pathogen infection assay was performed as described previously in cucumber [60]. Briefly, the second true leaves of four-week-old cucumber seedlings were sprayed with 10^6^ cfu/mL of *Psl*, samples were collected at pre-inoculation (0 day) as control, and 1, 2, 3 dpi for expression analysis. 

For analysis of *Foc*-inducible gene expression, the roots of one-week-old cucumber seedlings were dipped in *Foc* spore suspension (1 × 10^6^ spores/mL) as described previously [56]. Similarly, root samples were harvested on 0 (as control), and 2, 4, and 6 dpi for further expression analysis.

### 4.7. Subcellular Localization Analysis

The full-length coding sequences without stop codon of *CsExo70A1*, *CsExo70B*, and *CsExo70G1* were amplified and cloned into pSuper-1300-eGFP vector. Then the resultant vectors were transformed into agrobacterium strain GV3101 and infiltrated into *N. benthamiana* leaves as previously described [61]. After 72 h infiltration, the fluorescence signals of GFP were observed using a confocal microscope (Zeiss LSM880, Jena, Germany) at an excitation wavelength of 488 nm and an emission wavelength of 510 nm. All primers used for subcellular localization analysis are listed in Table S1. 

## 5. Conclusions

A total of 18 CsExo70 members were identified in this study. Tissue expression analyses showed that most *CsExo70* genes (except for *CsExo70E2* and *CsExo70H3*) exhibited a similar expression pattern, and *CsExo70C1* and *CsExo70C2* were highly expressed in the stamen. In addition, the expression levels of most *CsExo70* genes were induced by pathogens, especially *CsExo70E2* and *CsExo70H3*. Together, our results provide the basis for studying the functions of Exo70 members in cucumber.

## Figures and Tables

**Table 1:** Information of the Exo70 family in cucumber.

Gene Name	Gene ID	CDS ^1^	AA ^2^	MW (Da) ^3^	PI ^4^	Group
CsExo70A1	CsaV3_5G035240.1	1956	651	73573.72	8.37	Exo70.3
CsExo70A2	CsaV3_6G041070.1	1917	638	72857.09	6.95	Exo70.3
CsExo70B	CsaV3_1G036490.1	1905	634	72173.20	5.13	Exo70.2
CsExo70C1	CsaV3_4G002930.1	2025	674	76816.14	5.97	Exo70.2
CsExo70C2	CsaV3_5G024390.1	2115	704	81346.62	4.95	Exo70.2
CsExo70D	CsaV3_3G030800.1	1833	610	69471.55	5.38	Exo70.2
CsExo70E1	CsaV3_4G009180.1	1980	659	75539.35	4.94	Exo70.2
CsExo70E2	CsaV3_1G005990.1	1908	635	72952.51	6.17	Exo70.2
CsExo70F1	CsaV3_1G045240.1	1959	652	74088.89	5.00	Exo70.2
CsExo70F2	CsaV3_1G009630.1	1914	637	72342.77	5.16	Exo70.2
CsExo70G1	CsaV3_2G031330.1	2049	682	77109.98	8.87	Exo70.1
CsExo70G2	CsaV3_7G033810.1	2025	674	76901.53	6.67	Exo70.1
CsExo70H1	CsaV3_5G006610.1	1860	619	69563.75	5.75	Exo70.2
CsExo70H2a	CsaV3_6G022100.1	1896	631	71359.32	6.71	Exo70.2
CsExo70H2b	CsaV3_1G033630.1	1860	619	70481.59	5.83	Exo70.2
CsExo70H3	CsaV3_3G035190.1	1926	641	72726.64	5.95	Exo70.2
CsExo70H4	CsaV3_4G024610.1	1749	582	66156.59	6.14	Exo70.2
CsExo70I1	CsaV3_2G026060.1	2109	702	80192.32	5.69	Exo70.1

Note: ^1^: Coding sequence; ^2^: Length of the amino acid sequence; ^3^: Molecular weight; ^4^: Isoelectric point.

## Data Availability

Data are contained within this article and Supplementary Material.

## Supplementary Materials

The supporting information can be downloaded at: https://www.mdpi.com/article/10.3390/ijms241310929/s1.
