# Supplementary material for: Genome-Wide Analysis of Exocyst Complex Subunit Exo70 Gene Family in Cucumber
Source: Int J Mol Sci. 2023 Jun 30;24(13):10929. doi: 10.3390/ijms241310929 (PMC10342120; doi:10.3390/ijms241310929)
Supplement: Supplementary file 1 [file ijms-24-10929-s001.zip › ijms-2441705-supplementary.pdf]

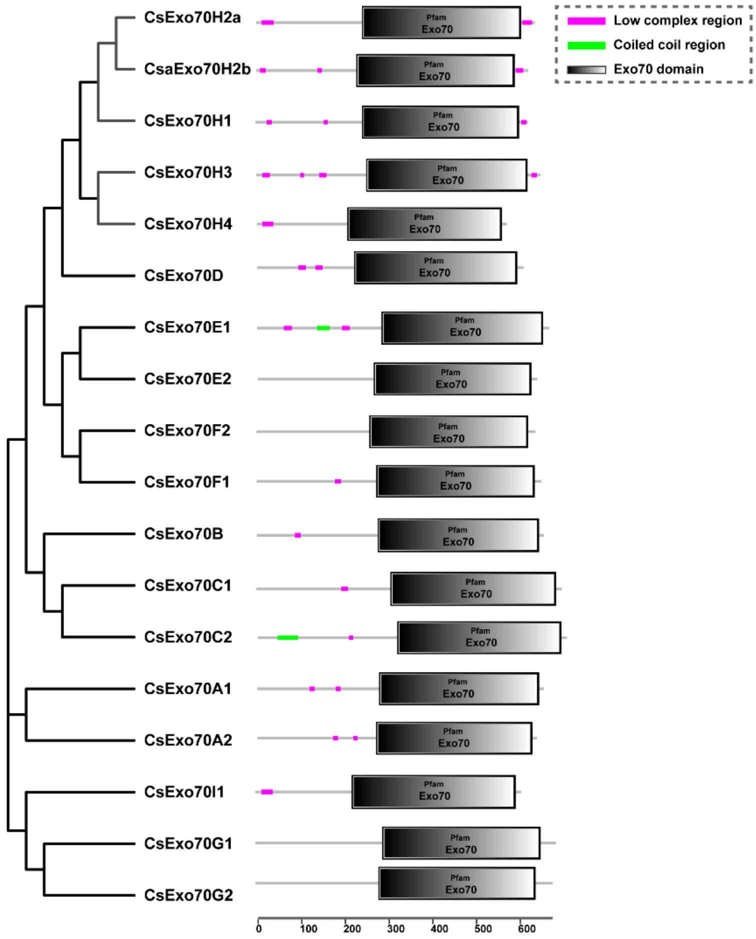

Figure S1. Conserved domains of CsExo70 members in cucumber. The amino acid sequences of all CsExo70s were downloaded from cucumber genome database (v3), and conserved domains were searched via SMART program.

Table S1. List of primers used in this study

**Primers for qRT-PCR**	
CsEXO70B-Q-F	5'-TCCGGGACTATCAACGATCT-3'
CsEXO70B-Q-R	5'-TCCCTCCTGCAGCTACTATAA-3'
CsEXO70C1-Q-F	5'-GAAGAAGACCGACCATCAAGAA-3'
CsEXO70C1-Q-R	5'-GCGTCGAGTAGTTCCATTACC-3'
CsEXO70C2-Q-F	5'-CGATTCTGATCCTGCTACTGATAC-3'
CsEXO70C2-Q-R	5'-CTCCTTGGAGTAACCTGGAAAC-3'
CsEXO70D-Q-F	5'-CGCAGAGGCGATCAGTATAAG-3'
CsEXO70D-Q-R	5'-GTCAGGTATCAACTCCGTCAAG-3'
CsEXO70E1-Q-F	5'-CCGTGACTCCCTCAATTTACTC-3'
CsEXO70E1-Q-R	5'-GAACTCACCTTCTCTATCTTCTTCTC-3'
CsEXO70E2-Q-F	5'-GGGTGGATGCGAGAACATATAA-3'
CsEXO70E2-Q-R	5'-CCATCAAGCCTTAGCAGAGAA-3'
CsEXO70F1-Q-F	5'-TGGATATGTACGACGCTTTGG-3'
CsEXO70F1-Q-R	5'-TTCACCCAGTCCACACAATAC-3'
CsEXO70F2-Q-F	5'-CTGACGCCGTTGTTGATTTG-3'
CsEXO70F2-Q-R	5'-CTAAAGCATCCCGACGAACA-3'
CsEXO70G1-Q-F	5'-GCCGAGATTGGAGGAGATTAG-3'
CsEXO70G1-Q-R	5'-GCCAACAGCCCGATTTATATG-3'
CsEXO70H3-Q-F	5'-GGAACCCTAGACGATGAAATCC-3'
CsEXO70H3-Q-R	5'-TGGCTCGTAAATCCTCCATAAC-3'
CsEXO70I-Q-F	5'-TGGACGACATGCGATTACAG-3'
CsEXO70I-Q-R	5'-GGAGTACGTCGCTCTATGTTTC-3'
CsUBQ-Q-F	5'-CACCAAGCCCAAGAAGATC-3'
CsUBQ-Q-R	5'-TAAACCTAATCACCACCAGC-3'
**Primes for subcellular localization**	
CsExo70A1-pSuper-F	5'-caaatcgactctagaaagcttATGGGTGTTCCTGCAACTGC-3'
CsExo70A1-pSuper-R	5'-gcccttgctcaccatggtaccCCGTTTAGGTTCATTCACATTCTTC-3'
CsExo70B-pSuper-F	5'-caaatcgactctagaaagcttATGGTTGACAATGGCGAAGAA-3'
CsExo70B-pSuper-R	5'-gcccttgctcaccatggtacc CCTCCTTCCACTGCCTGA-3'
CsExo70G1-pSuper-F	5'-caaatcgactctagaaagcttATGACGGCACCGGTGTCC-3'
CsExo70G1-pSuper-R	5'-gcccttgctcaccatggtaccCATAACCATGGAGTTGGAACGA-3'

Note: The lower letters represent the vector sequence and the red represents the restriction site of the vector.
